# The Importance of Group-Wise Registration in Tract Based Spatial Statistics Study of Neurodegeneration: A Simulation Study in Alzheimer's Disease

**DOI:** 10.1371/journal.pone.0045996

**Published:** 2012-11-06

**Authors:** Shiva Keihaninejad, Natalie S. Ryan, Ian B. Malone, Marc Modat, David Cash, Gerard R. Ridgway, Hui Zhang, Nick C. Fox, Sebastien Ourselin

**Affiliations:** 1 Dementia Research Centre, University College London Institute of Neurology, London, United Kingdom; 2 Centre for Medical Image Computing, Department of Medical Physics and Bioengineering, University College London, London, United Kingdom; 3 Wellcome Trust Centre for Neuroimaging, University College London Institute of Neurology, London, United Kingdom; Banner Alzheimer's Institute, United States of America

## Abstract

Tract-based spatial statistics (TBSS) is a popular method for the analysis of diffusion tensor imaging data. TBSS focuses on differences in white matter voxels with high fractional anisotropy (FA), representing the major fibre tracts, through registering all subjects to a common reference and the creation of a FA skeleton. This work considers the effect of choice of reference in the TBSS pipeline, which can be a standard template, an individual subject from the study, a study-specific template or a group-wise average. While TBSS attempts to overcome registration error by searching the neighbourhood perpendicular to the FA skeleton for the voxel with maximum FA, this projection step may not compensate for large registration errors that might occur in the presence of pathology such as atrophy in neurodegenerative diseases. This makes registration performance and choice of reference an important issue. Substantial work in the field of computational anatomy has shown the use of group-wise averages to reduce biases while avoiding the arbitrary selection of a single individual. Here, we demonstrate the impact of the choice of reference on: (a) specificity (b) sensitivity in a simulation study and (c) a real-world comparison of Alzheimer's disease patients to controls. In (a) and (b), simulated deformations and decreases in FA were applied to control subjects to simulate changes of shape and WM integrity similar to what would be seen in AD patients, in order to provide a “ground truth” for evaluating the various methods of TBSS reference. Using a group-wise average atlas as the reference outperformed other references in the TBSS pipeline in all evaluations.

## Introduction

The analysis of diffusion weighted MRI (DWI) data has become an increasingly important area of neuroimaging research. DWI contains information for assessing white matter (WM) integrity, architecture and connectivity patterns. Diffusion tensor imaging (DTI), in particular, describes the local diffusion process or the 3D probability profile of water diffusion in tissue. One approach to quantifying white matter structure using DTI data is to compute scalar summaries such as fractional anisotropy (FA), mean, axial and radial diffusivity [1], [2]. Mapping these parameters enables investigation of pathological change in the cerebral white matter.

Tract-Based Spatial Statistics (TBSS) is an automated method for DTI analysis that employs a voxel-wise comparison-based approach to assess associations across subjects, e.g. differences between groups [3]. TBSS alleviates the alignment-related problems of the low-resolution DTI data by projecting the FA values of individual subjects onto a common “FA-skeleton” of major white matter structures. This process is done through linear and non-linear alignment, thus improving interpretability of analysis of multi-subject DTI data [3]. However, this mapping may not cope with high inter-individual brain variability, especially in the presence of cerebral atrophy and ventricular expansion observed in aging, and to a much greater extent in neurodegenerative disorders such as Alzheimer's disease (AD). These structural changes can cause difficulties in aligning images to a predefined atlas, particularly if the atlas has been generated from the brain scans of young, healthy volunteers. The choice of a reference image can strongly impact the results and the interpretation of statistical comparisons between cohorts [4]. Some studies have attempted to overcome the misalignment problem by modifying the registration step in the TBSS pipeline. For example, to handle substantial ventricular enlargement in a study of AD patients, Douaud et al. created a study-specific FA template by non-linearly registering all native-space FA images to an FA template in the MNI space (www.fmrib.ox.ac.uk/fsl/data/FMRIB58_FA) and then averaging them [5]. Then, the original FA scans were non-linearly registered to this study-specific FA template. This registration is still essentially pairwise from each image to the reference FMRIB58FA, constructed from 58 FA maps acquired from healthy adults aged 20–50 years. Although the original FA scans were non-linearly registered to this study-specific FA template rather than the atlas, it is still dependent on the registration performance with respect to the atlas. To this end, we propose incorporating a group-wise atlas into the TBSS pipeline which, to the best of our knowledge, has not been previously implemented.

In order to validate the skeleton projection algorithm at the heart of TBSS, Zalesky presented an evaluative methodology using synthetic warps of a ground truth image to quantitatively assess TBSS performance based on three healthy subjects and two sets of FA images [6]. This study suggested that even though the skeleton projection only recovers less than 10% of the post-registration misalignment, it still resulted in far less error of the expected FA value than using Gaussian smoothing to reduce the effects of misregistration. However, no work has been done, to our knowledge, to evaluate the performance of TBSS when there is a large deformation present due to atrophy. We extended the performance evaluation to simulate morphometric variation similar to neurodegeneration in AD.

## Materials and Methods

**Figure 1:**
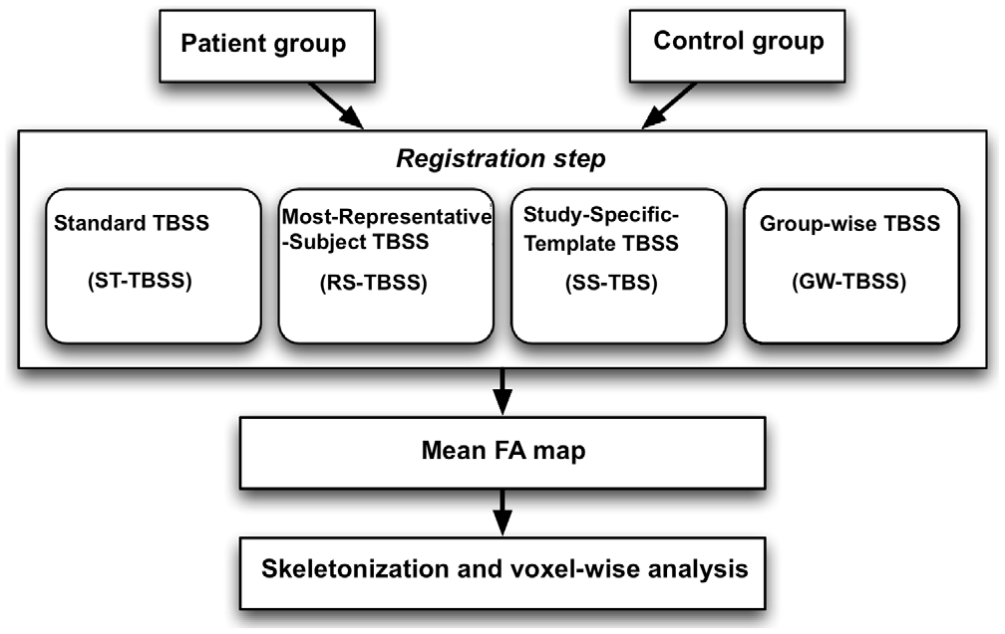
TBSS processing pipeline. The focus of this study is on the non-linear registration step which is investigated using four different approaches. Registration steps are described in Section.

The TBSS pipeline for the studies in this paper, depicted in Figure 1, consists of the following steps:

1. Registration: every FA image is registered to a FA target image in standard space.
2. Mean FA: aligned FA images are averaged to create a mean FA map.
3. Skeletonization: a white matter skeleton is created, representing major tracts common across all subjects. A threshold of FA>0.2 is set to include the major white matter pathways, but to exclude peripheral tracts where there was significant inter-subject variability and/or partial volume effects with grey matter.
4. Projection step: TBSS then projects each subject's FA data onto the mean WM tract skeleton. The highest FA value near the skeleton in each subject (which should correspond to the local tract centre value) is then projected onto the mean WM tract skeleton for analysis.
5. Voxel-wise statistics: Voxel-wise statistical analyses were performed by using a permutation-based inference tool for nonparametric statistical thresholding (“randomise” program, part of FSL [7]) to assess group-related differences. The number of permutations was set at 5000 [7]. This method delivers non-parametric, two-sample, unpaired t-tests of reduced and increased DTI indices in patients compared with controls. TBSS results for FA was considered significant for *P*<5, corrected for multiple comparisons using threshold-free cluster enhancement (TFCE), a method which avoids using an arbitrary threshold for the initial cluster-formation [8].

In this study, we wished to examine the specific effects of the registration step. In particular we aimed to investigate and compare different approaches in the non-linear registration step of the TBSS pipeline by designing a simulation study.

### Registration procedures

For consistency and repeatabilty purposes, all linear registrations in this study were performed using FMRIB's linear image registration tool (FLIRT) [9]. Once aligned using linear registration, the non-linear registrations were then performed using FMRIB's Non-Linear Registration Tool (FNIRT), with the parameters as defined in FA_2_FMRIB58_1 mm configuration file [10].

**Figure 2:**
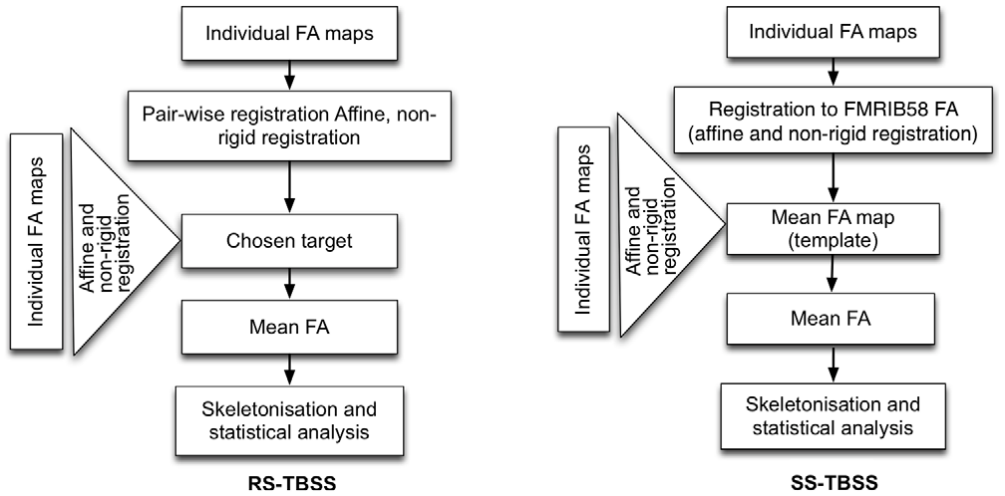
Most-Representative-Subject TBSS (RS-TBSS) and Study-Specific-Template TBSS (SS-TBS) pipeline. The remainder of this paper is organised as follows: In Section, different registration approaches for the TBSS pipeline are reviewed and a modification to the pipeline is introduced to incorporate a group-wise atlas. In Section, a misalignment between two groups (patients and controls) is modelled using a simulation study. In Section, results are presented on the simulation study and on a dataset of AD (n = 20) and age-matched controls (n = 21).

The common TBSS pipeline provides three different routes for the registration step:

• Standard TBSS (ST-TBSS): All subjects are directly aligned, linearly and then non-linearly, to the standard space FA template (FMRIB58 FA).
• Most-Representative-Subject TBSS (RS-TBSS): In this method, the most representative FA image is chosen by first performing all possible pairwise registrations (both linear and non-linear) between subjects. From this, the subject that has the minimum mean deformation required to non-linearly align it to all the subjects will serve as the reference. This target image is then affine-aligned into standard space, and every image is transformed into FMRIB58
- FA space by combining the nonlinear transform to the target FA image with the affine transform from that target to standard space. Figure 2 provides a diagram of this process.
• Study-Specific-Template TBSS (SS-TBS): As mentioned above, the subjects are all aligned (linearly and non-linearly) in standard space to the FMRIB58
- FA template; averaged to give a morphometric average atlas, (Mean FA map template in Figure 2); then the original FA images are non-linearly registered to this specific FA template (pre-defined target) [5].

Table 1 summarises studies of AD which have utilised TBSS to date.

**Table 1:** Anatomical locations reported to show reduced FA in AD patients in the literature using TBSS to date.

Authors, Year	Subjects	Method	Areas of reduced FA
	(age: Mean ± SD)		
[**26**]	19 Control (75.0±6.0)17 AD (76.0±7.0)	ST-TBSS	Parahippocampus WM (right), uncinate fasciculus (bilateral), WM tracts in brain stem and cerebellum, inferior and superior longitudinal fasciculus, cingulum, corpus callosum (genu and splenium; no change in body and Rostrum), fornix and cerebellum (*p*<0.01, uncorrected).
[**29**]	22 controls (70.0±6.0) 16 AD (69.5±6.7)	ST-TBSS	The medial temporal white matter and uncinate fasciculus (*p*<0.0001 corrected).
[**27**]	54 Control (75.8±5.6)20 AD (77.8±4.9)	ST-TBSS	Lateral occipital, middle and inferior temporal WM, inferior parietal/supramarginal, precuneus and parahippocampal WM
[**30**]	13 controls (64.1±10.5)9 AD (72.4±7.5)	ST-TBSS	Corpus callosum (splenium), right fornix, right cingulum, anterior thalamic radiations (bilaterally), Inferior longitudinal fasciuclus (bilaterally) and right posterior thalamic radiation (*p*<0.05 corrected).
[**25**]	15 controls (75.2±5.6)15 AD (72.2±5.7)	ST-TBSS	Posterior areas of the left hemisphere, in anterior areas, the left uncinate fasciculus, left inferior fronto-occipital and cingulate bundles, in temporal, parietal and occipital regions, parts of the inferior fronto-occipital, inferior longitudinal, superior longitudinal and cingulate tracts (*p*<0.05 corrected).
[**24**]	15 control (74.1±6.1)15 AD (75.27±3.1)	ST-TBSS	Posterior left hemisphere involving the uncinate fasciculus and inferior fronto-occipital and cingulate bundles (*p*<0.05 corrected).
[**31**]	22 controls (70.7±6.0)16 AD (69.5±6.9)	RS-TBSS	Anterior part of the left temporal lobe, probably in the uncinate fasciculus (*p*<0.05 corrected).
[**22**]	13 controls (67.1±5.5)25 AD (69.7±6.3)	RS-TBSS	Right temporal lobe, right posterior cingulate region, right parieto-occipital region, fornix as well as two small areas in the right cerebellar hemisphere and ponto-medullary junction (*p*<0.01 uncorrected for multiple comparisons).
[**23**]	15 control (69.8±6.0)23 AD (74.6±8.6)	RS-TBSS	Parahippocampal tract, fornix, and small, inferior parietal regions (*p* = 0.05 uncorrected).
[**28**]	14 controls (77.3±9.0)16 AD (77.4±8.1)	RS-TBSS	Uncinate fasciculus, inferior longitudinal fasciculus, superior longitudinal fasciculus, limbic pathways (fornix/stria terminalus, cingulum), and commissural pathways.
[**5**]	61 controls (71.1±8.3)53 AD (74.1±8.6)	SS-TBSS	Corpus callosum, anterior commissure, uncinate fasciculus, cingulum bundle and superior longitudinal fasciculus (*p*<0.05 corrected).

In some, a mild cognitive impairment (MCI) group was studied alongside the AD and control groups. For simplicity we summarise the FA findings of the AD versus control group comparison only. ST-TBSS: Standard; RS-TBSS: Most-Representative-Subject TBSS; SS-TBSS: Study-Specific-Template.

#### Group-wise TBSS (GW-TBSS)

The key idea explored in this study is to define a group-wise atlas and incorporate it into the TBSS pipeline. Several studies have used a study-specific brain template in voxel-based morphometry (VBM) [11], [12]. Rose et al. expanded the use of VBA-type analysis by using study-specific averages in order to investigate mean diffusivity and FA changes in both grey and white matter structures in patients with AD [13]. However, to the best of our knowledge, none of the TBSS based studies have used a group-wise atlas which serves as the fourth and novel option in this study.

An approach based on [14] was used to create the group-wise atlas image. It consisted of a two-step method, where the first step consists of registering all of the input images to the atlas image and the second step corresponds to the update of the atlas image. This process is repeated until the atlas image converges. In this study, we used a coarse-to-fine approach where the deformation model for registration was first rigid, then affine and finally non-rigid. The atlas image was initialised as a random image from the dataset, with all updates at the end of each iteration corresponding to the average of all registered images. Note that the first registration step only consists of rigid registration in order that no bias was introduced from the random selection of the initial image as the atlas. Five iterations were performed using a global registration (one rigid and four affine) and then ten iterations of non-rigid registration. Once the atlas was created, it was registered, through affine registration, to FMRIB58FA. We then used the transformation to align each input FA image to FMRIB58FA.

### Experiments

All registration methods were evaluated on a dataset of 41 subjects: 20 AD patients and 21 controls matched for age and gender. All of the patients had attended the Cognitive Disorders Clinic at the National Hospital for Neurology and Neurosurgery, London, where they had been diagnosed clinically with AD of mild to moderate severity. Informed consent was obtained from all subjects and the study had local ethics committee approval. Subject demographics and mini-mental state examination (MMSE) scores are shown in Table 2.

**Table 2:** Demographic and clinical data for the Alzheimer's disease (AD) patients and healthy control subjects whose scans were used in this study.

	Age (years)	Gender	MMSE	Disease duration in years
	Mean (sd)	M/F	Mean (sd)	Mean (sd)
AD (n = 20)	61.3 (4.8)	7/13	16.0 (5.5)	5.9 (2.3)
Control (n = 21)	61.2 (7.3)	8/13	29.6 (0.5)	N/A
Control* (n = 10)	63.1 (5.1)	3/7	30.0 (0.5)	N/A

* : Control subjects in simulation study.

Ethical approval for the study was received from the Joint Ethics Committee of The Institute of Neurology and the NHNN (National Hospital for Neurology and Neurosurgery). All subjects gave written informed consent according to the Declaration of Helsinki. Consent was taken by a clinician experienced in the assessment of patients with cognitive impairment and all subjects were considered to have capacity to consent according to the Mental Capacity Act of 2005.

Each subject was scanned on a Siemens Tim Trio 3 Tesla scanner using a 32-channel head coil. Diffusion weighted images were acquired, TR = 6500 ms, TE = 83 ms, 2.5 mm isotropic voxels, 9696 acquisition matrix and 55 slices, with two sets of 64 direction diffusion gradients (diffusion weighting 1000 ) and nine unweighted volumes. Images were affinely, registered to the first unweighted volume with FLIRT to correct for motion and eddy currents and the weighting vectors adjusted for rotation. Diffusion tensors were fitted with the Camino package [15] using all acquired volumes.

### Specificity evaluation

The obstacle faced in devising a performance measure of TBSS is that the full knowledge of the ground truth is unavailable. To overcome this obstacle we used a similar approach to [6]. The misalignment was artificially modelled by warping the FA images of ten control subjects using a deformation field designed to model the typical deformation pattern observed in AD. The warped controls served as the second group for comparison. If the registration strategy of TBSS is suitably compensating for the alignment, then no significant differences should be identified through the voxel wise statistical analysis performed by TBSS. Any significant clusters observed were considered false positives.

**Figure 3:**
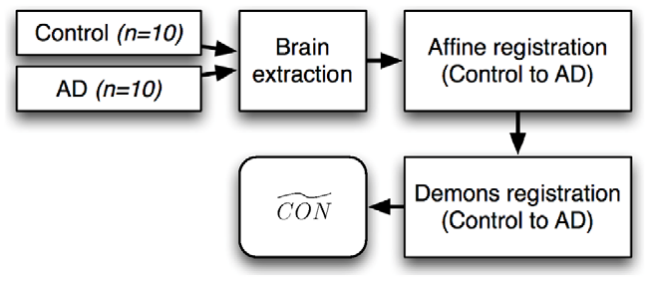
Flowchart of the simulation study. Flowchart of creating the misalignment used in the specificity evaluation. Ten control images (*CON*) images were individually warped to 10 AD images to produce ten warped control images using ITK-based affine registration method and Demons.

**Figure 4:**
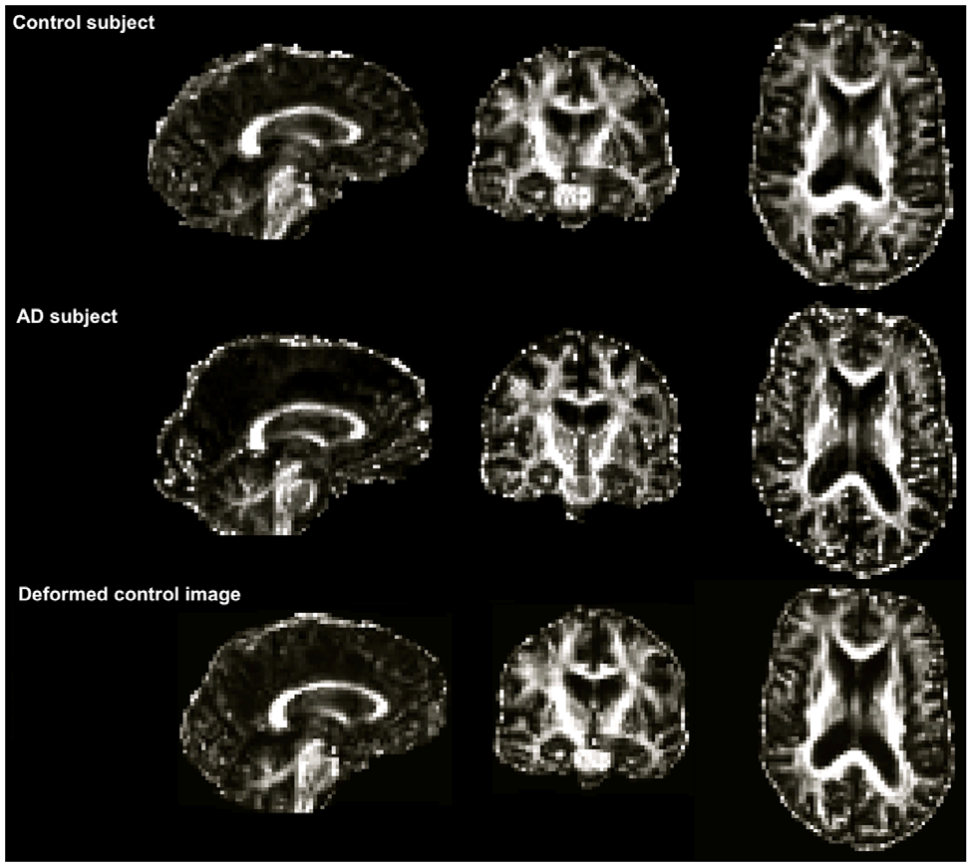
Modelling typical atrophy in AD using Demons registration algorithm. A control image, *CON*, an AD subject as the target and the deformed control image, , after applying the registration. Ventricular expansion in AD is well modelled in the control subject using the Demons algorithm.

Figure 3 shows the flowchart for creating the misalignment used in the specificity evaluation. Ten control images (*CON*) images were individually warped to 10 AD images to produce ten warped control images using a registration pipeline independent to the methods used in the registration step of the TBSS pipeline. All FA images were first skull stripped using Brain Extraction Tool (BET) [16]. Then, each control-AD image pair was aligned linearly using an ITK-based affine registration method [17] with the normalised cross-correlation as the similarity measure and trilinear interpolation. After affine alignment, a non-linear registration was performed using an ITK-based method, Demons, applied with default parameters [18]. This non-parametric algorithm has been successfully applied to DTI data [19]. The Demons method was chosen for the artificial warp to avoid bias toward the deformation model, as it is a non-parametric approach while we use a parametric registration algorithm (FNIRT) within the TBSS pipeline. Figure 4 shows an example control image, the AD subject it is being registered to, and the resulting warped control image. All resampling of images was performed using trilinear interpolation. To avoid detecting any differences introduced by the interpolation scheme, the *CON* images were smoothed with a Gaussian kernel in an attempt to match the level of smoothing caused by the interpolation. Each was mapped back to the corresponding *CON* using the inverse transformation and then the root-mean-square error (RMSE) was computed. The *CON* images were smoothed with a Gaussian kernel, when the widths of kernel () varying 0.5,1,1.5 and 2 mm. A kernel width of 1.5 mm was chosen based on the minimum average RMSE obtained between the smoothed CON and .

False-positive error is measured based on the voxels that show significant statistical difference between two groups even though there is no difference between them.

### Sensitivity evaluation

**Figure 5:**
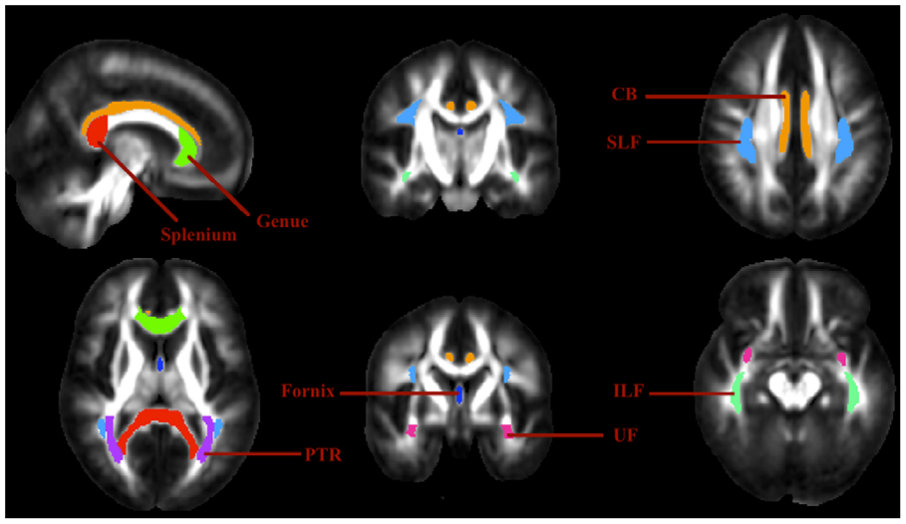
WM tract masks used in the true-positive experiment. CB: Cingulum bundle; ILF: Inferior Longitudinal fasciculus (including the Inferior fronto-occipital fasciculus); SLF: Superior longitudinal fasciculus; UF: Uncinate fasciculus; PTR: Posterior thalamic radiation.

In addition to the specificity evaluation, we performed a sensitivity evaluation to investigate the performance of the various TBSS strategies when there is a true difference between groups. For this, the FA values of voxels in the WM tracts listed in Table 3 were changed in the () group. The ICBM-DTI-81 WM labels atlas within FSL, developed by Johns Hopkins University (JHU), was used to locate the WM tracts of interest [20]. Each individual FA image was linearly registered to the atlas space using an affine registration. After affine registration, the ICBM WM atlas was non-linearly registered to the FA images in template space using FNIRT. This transformation was used to warp the labels from the ICBM WM atlas to the individual FA image through nearest neighbour interpolation. The anatomical locations of the WM tracts in Table 3 were produced (Figure 5) and checked by an experienced neuroradiologist. The FA values within the WM masks were then reduced by 10%, 20%, 30% and 40% in the group synthetically. The resulting new and original FA images (*CON*) were processed through the TBSS pipeline.

**Table 3:** Summary of the results obtained with different TBSS pipelines in the literature and specificity evaluation study on FA.

White matter tracts	No. studies			specificity evaluation			
	ST-TBSS	RS-TBSS	SS-TBSS	ST-TBSS	RS-TBSS	SS-TBSS	GW-TBSS
	n = 6	n = 4	n = 1				
Uncinate fasciculus	4	1	1	√ (×)	√ (×)	×^3^ (×)	× (×)
Inferior longitudinal fasciuclus	3	1	–	√ (×)	√ (×)	√ (×)	× (×)
Superior longitudinal fasciculus	2	–	–	× (×)	√ (√)	√ (×)	× (×)
Cingulum bundle	5	1	1	√ (√)	√ (√)^1^	√ (×)	× (×)
Genu (CC)	1		1	√ (×)	√ (√)	√ (×)	× (×)
Splenium (CC)	2	–	1	√ (√)	√ (√)	√ (×)	× (×)
Fornix	2	2	1	√ (×)	√ (×)	× (×)	× (×)
Anterior thalamic radiations	1	–	–	√ (×)	√ (√)	× (×)	× (×)
posterior thalamic radiation	1	–	–	× (×)	× (×)	× (×)	× (×)
Inferior fronto-occipital	2	–	–	√ (×)	√ (√)^2^	√ (×)	× (×)
WM of the parahippocampal gyrus	2	1	–	√ (√)	√ (√)	√ (×)	× (×)

significant reduction in FA (); no significant results; n = number of studies.

CC: Corpus callosum; ST-TBSS: Standard; RS-TBSS: Most-Representative-Subject TBSS; SS-TBSS: Study-Specific-Template; GW-TBSS: Group-wise TBSS; Results of the specificity evaluation study is reported bilaterally and in the case of asymmetry they are reported for the right hemisphere. 1: Left with only significant difference in ; 2: Left with no significant difference; 3: Left with significant difference at .

### TBSS analysis of pathology

The TBSS pipeline was applied to the full data set of the 21 controls and 20 AD patients with each of these different approaches: Standard (ST), Most-Representative-Subject (RS), Study-Special-Template (SS) and Group-wise (GW) TBSS.

## Results

### Specificity evaluation

Table 3 shows the results from the specificity evaluation study. ST-TBSS shows significant difference at a corrected level in all regions, except for the posterior thalamic radiation and superior longitudinal fascicles. These significant differences should be interpreted as likely false positives. Only the cingulum bundle and WM of parahippocampal tract were significant at . These results were bilateral.

The RS-TBSS method showed higher false positives in more WM tracts with less symmetrical results. SS-TBSS showed less significant differences compared with the Standard pipeline, suggesting that an atlas specifically created from the study can help to reduce the misalignment problem. GW-TBSS showed no difference between the two groups in the listed WM tracts (Table 3).

**Figure 6:**
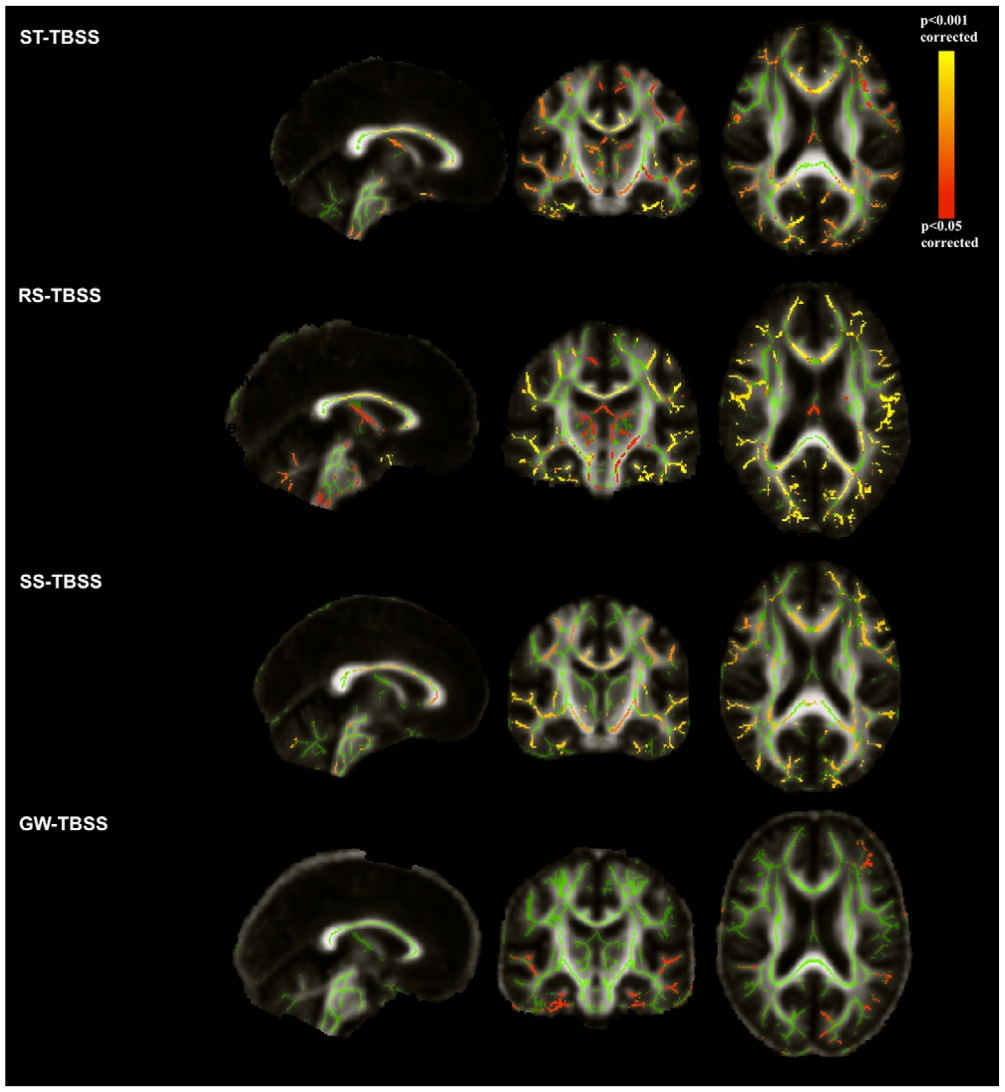
TBSS contrasts between two control groups (*CON* and) using different registration schemes. The contrasts are overlaid on the mean FA map of each approach and the mean FA skeleton (in green, FA 0.2). The results are thresholded at , corrected for multiple comparisons. The yellow-red color indicate the areas with significantly decreased FA values in deformed control images compared with the original controls.

Figure 6 shows the spatial distribution of significant differences (representing false positives) in FA between and *CON*. In ST, RS and SS-TBSS, regions of false-positive error are evident in the statistical map of the brain. These changes can occur due to residual misalignment. Although SS-TBSS helped to reduce the amount of false positive regions, some areas still remain in multiple WM tracts with less significant values than in the ST-TBSS method (Figure 6). The voxel-wise statistical map resulting from the GW-TBSS method has fewer false-positive regions of difference.

As a reference, Table 3 also summarises the findings from the literature regarding the WM tracts that were reported to demonstrate significant reduction of FA in AD (it should be noted that some studies only specified large regions instead of specific WM tracts). The findings of the studies in the literature vary not only when different TBSS approaches were used, but also when the same TBSS approach was used. This is often attributed to differences in the study samples, but factors such as residual misalignment in the FA images may also contribute to the discrepancies.

### Sensitivity evaluation

Table 4 shows the results from the sensitivity evaluation study. GW-TBSS was able to detect the significant reduction of FA in all the examined WM tracts at every reduction level. There was no asymmetric difference when the FA values of the WM tracts were changed bilaterally. ST-TBSS and RS-TBSS detected the significant reduction in FA in all WM tracts at all reductions, except in the posterior thalamic radation at 10% reduction. SS-TBSS also detected all of the changes except for the fornix at 10%. Although these methods were highly sensitive, many of these tracts were identified as false positive in the specificity evaluation.

**Table 4:** Results obtained with Group-wise TBSS on sensitivity evaluation study when reducing FA virtually.

White matter tracts	sensitivity evaluation study			
	ST-TBSS	RS-TBSS	SS-TBSS	GW-√TBSS
Uncinate fasciculus	√(√)	√(√)	√(√)	√(√)
Inferior longitudinal and Inferior fronto-occipital fasciuclus	√(√)	√(√)	√(√)	√(√)
Superior longitudinal fasciculus	√(√)	√(√)	√(√)	√(√)
Cingulum bundle	√(√)	√(√)	√(√)	√(√)
Corpus callosum (Genu)	√(√)	√(√)	√(√)	√(√)
Corpus callosum (Splenium)	√(√)	√(√)	√(√)	√ (√)
Fornix	√(√)	√(√)	×(√)	√ (√)
posterior thalamic radiation	×(√)	×(√)	√(√)	√ (√)

Corrected p-value at ; 10% (20–40%) FA reduction; significant reduction in FA at ; no significant results.

ST-TBSS: Standard; RS-TBSS: Most-Representative-Subject TBSS; SS-TBSS: Study-Specific-Template.

### Effect of the registration scheme on skeletonisation

**Figure 7:**
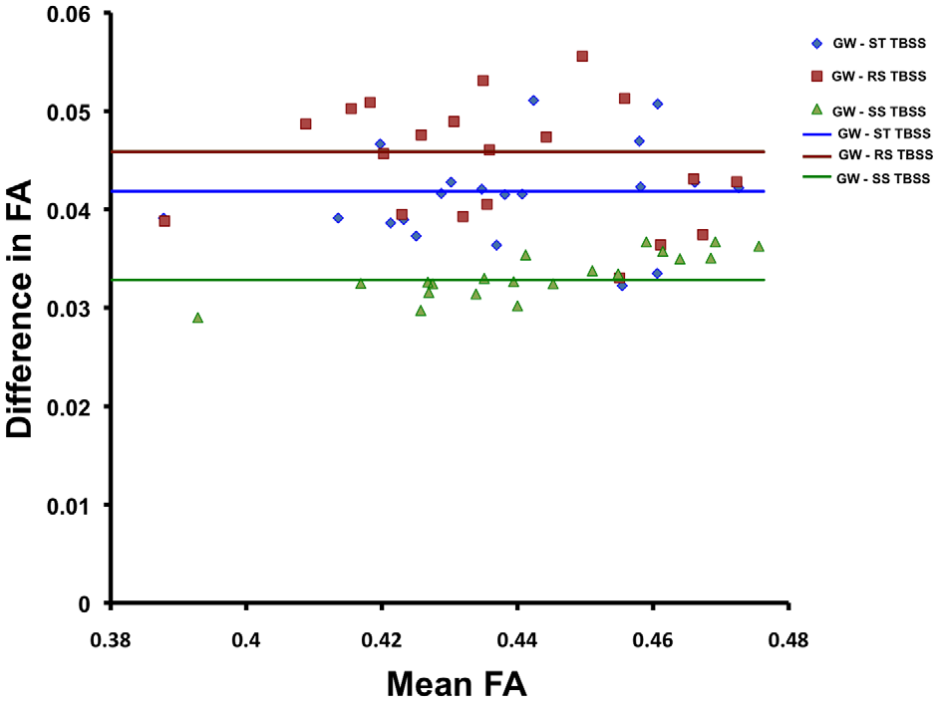
Bland-Altman plot showing differences in projected FA between GW-TBSS and the established methods of registration. GW-TBSS has a higher projected FA across the mean skeleton compared to ST-TBSS, RS-TBSS and SS-TBSS. Median difference in FA are shown with horizontal lines for each comparison.

To quantify the effect of the alignment on the skeletonisation and projection step of the TBSS protocol, Figure 7 shows a Bland-Altman plot of individual FA values extracted from the intersection of skeletonised datasets of GW-TBSS with other methods (Group-wise – other methods) in the specificity evaluation study. Projected FA was significantly higher across the whole skeleton after registration to the Group-wise atlas in comparison with the other methods.

### Effect of the registration scheme on variation of FA across the group

**Figure 8:**
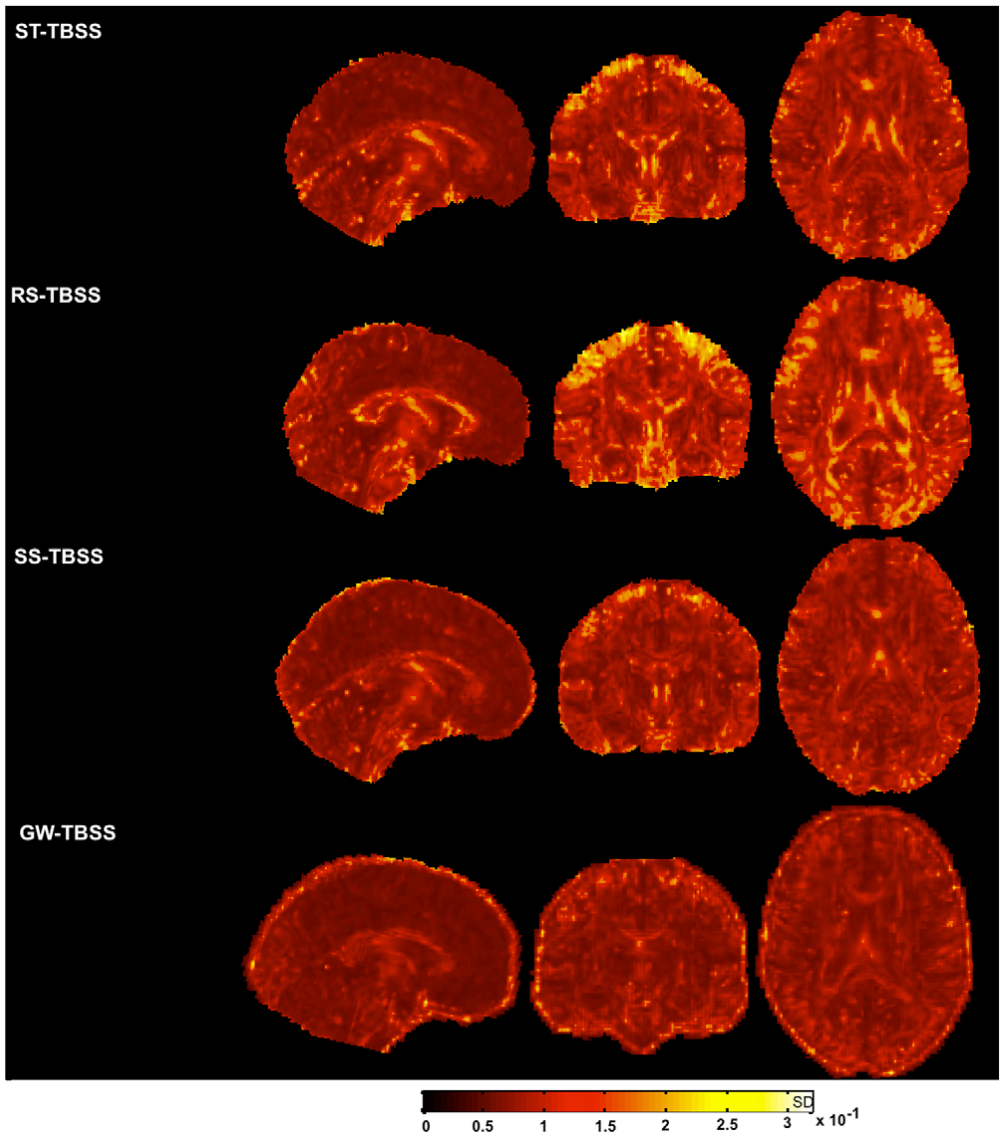
Standard deviation in FA across the group after registration to the FA template (FMRIB58_FA) in ST-TBSS, RS-TBSS, SS-TBSS, GW-TBSS. Standard deviation maps indicate standard deviation was greater when using ST-TBSS and RS-TBSS. Colour bar indicates standard deviation.

Figure 8 shows the voxel-wise standard deviation of FA across the group calculated after applying ST, RS, SS and GW-TBSS in and *CON*. Higher standard deviation is visible in several regions when using ST, RS and SS-TBSS such as around the ventricles and parts of the corpus callosum. Group-wise alignment reduces the inter-subject FA variance, suggesting that the FA images are better aligned.

**Figure 9:**
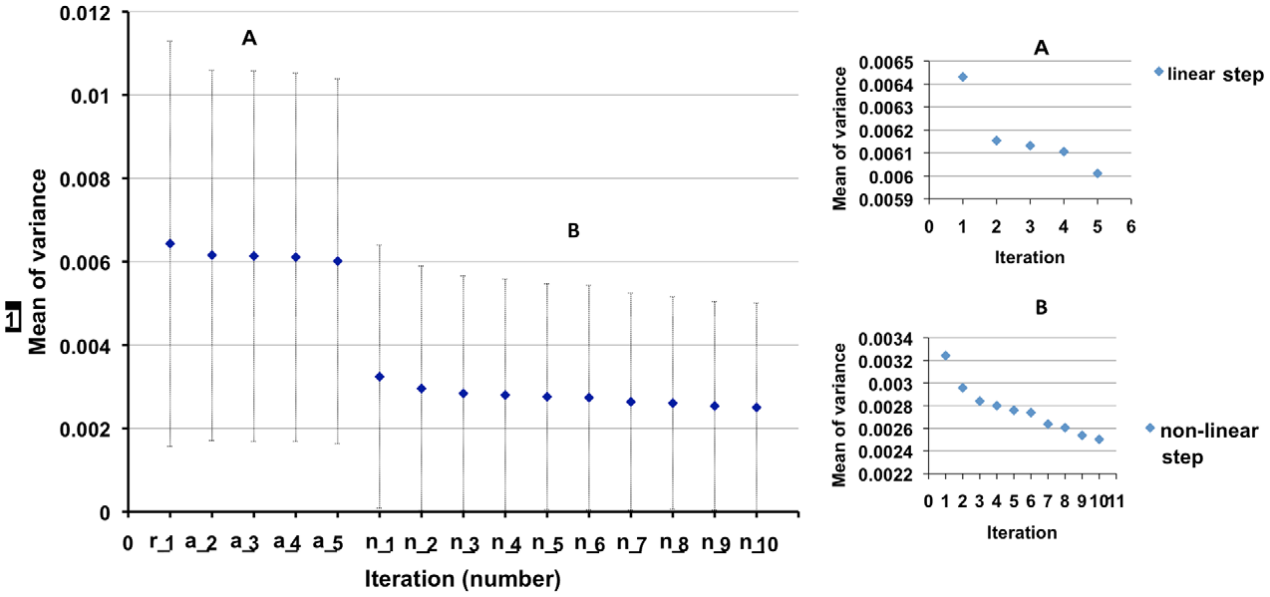
Mean variance of difference between average image and subjects in each iteration when using GW-TBSS. The mean and standard deviation of variance reduces in each iteration, r: rigid registration; a: affine registration; n: non-linear registration. Inlays of the linear (top right) and non-linear (bottom-right) iterations are shown separately to better illustrate the improvement of the group wise registration with each iteration.

Figure 9 shows that the mean (over voxels) of the variance of difference between the average image and subjects reduces with each iteration when using GW-TBSS. The mean of this variance of difference over subjects when using SS-TBSS (0.0041±0.0038) is higher than the first iteration of non-linear registration in GW-TBSS.

### TBSS analysis of AD pathology

**Figure 10:**
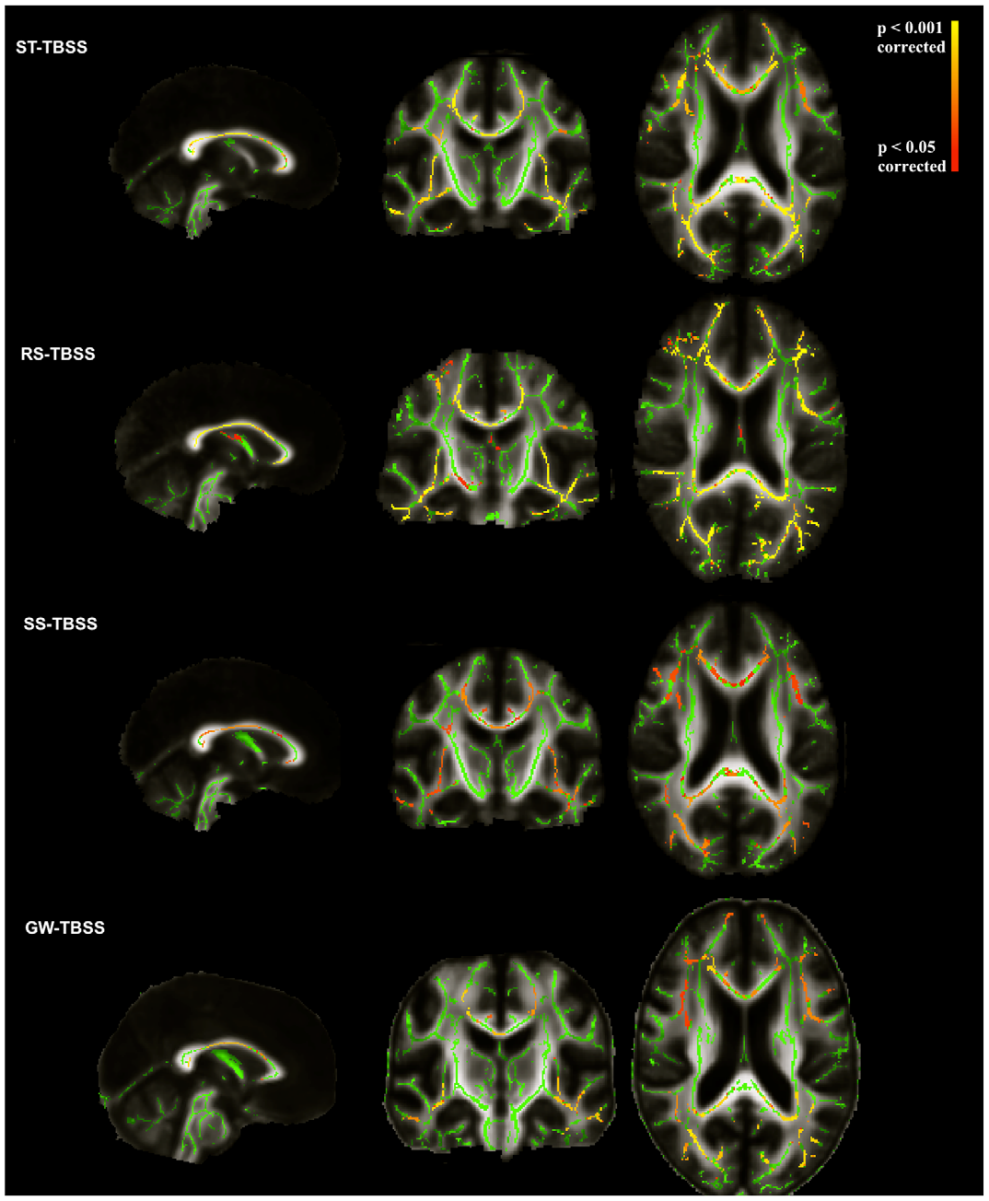
The voxel-wise statistical map between 20 patients with AD and 21 controls using different TBSS approaches. FA results showing the contrast ADCON; Statistical threshold: p0.05 (corrected); In green the mean FA skeleton is shown and the statistical maps are overlaid on the mean FA image of each approach.

Figure 10 shows the location of significant differences ( corrected) in FA between patients with AD and control subjects when using the TBSS pipeline with different approaches, including our GW-TBSS. Age and gender were included as covariates for the analyses as in [21]. The voxel-wise statistical map is overlaid on the mean FA map of each method.

Different TBSS approaches showed reduced FA in patients with AD in bilateral uncinate, inferior longitudinal and inferior fronto-occipital fasciculi and posterior thalamic radiation, right cingulum bundle, genu, body and splenium of corpus callosum. However, RS-TBSS showed significant FA reductions in widespread areas throughout the brain. Only SS-TBSS showed significant FA reduction in the left cingulum bundle. No significant FA reduction in the AD group was found using SS-TBSS and GW-TBSS for the fornix and WM of parahippocampal gyrus, whilst RS-TBSS showed significant FA reduction in both WM tracts and ST-TBSS showed FA reduction in WM of parahippocampal gyrus. Studies listed in Table 1 using different TBSS pipelines showed significant FA reductions of these WM tracts in AD groups.

## Discussion

The aim of using TBSS for analysis of diffusion data is to provide an objective and sensitive method for multi-subject, whole-brain diffusion data analysis. The registration step that forms a key part of the TBSS algorithm is designed to align each individual's FA image to a common standard space. Minimising residual misalignment is critical to ensure that the sensitivity and specificity of any subsequent statistical analysis is not compromised due to poor alignment [6]. The skeleton projection step is designed to alleviate residual misalignment following the registration step, however the projection procedure must search locally (to avoid finding spurious correspondences) so will not be able to correct large misalignment. One reason for such misalignment may be the inclusion of subjects with variable levels of cerebral atrophy and ventricular size. Given the widespread and growing use of TBSS in studying populations with atrophy, for example AD patients, the purpose of this study was to investigate alternative registration procedures for TBSS and explore the potential for a group-wise atlas to minimise false findings caused by misalignment.

### TBSS method in atrophy

Many studies have now applied TBSS to AD patients, with variable results (Table 1). Acosta-Cabronero et al. found no significant FA change when corrected for multiple comparisons [22]. Agosta and colleagues reported significant FA reduction limited to the parahippocampal tract and the fornix [23]. Numerous studies have reported significant FA reduction in widespread areas throughout the brain including all of the regions that were used in this paper [24], [25], [26], [27], [28], [29], [30], [5], [31] (see Table 3).

These studies are not directly comparable to each other due to differences in subjects (sample size, age range, disease severity), data acquisition protocols and statistical procedures, which may underlie some of the inconsistencies in their findings regarding affected WM tracts. However, it is very possible that methodological factors such as registration performance may have contributed in some way to the discrepancies. The performance of the registration algorithm may be degraded if the deformations required to transform one image into another are too large. Therefore, the statistical results obtained from a conventional TBSS pipeline (including ST and RS-TBSS) may be affected by the performance of the registration in the study group where atrophy is present. Use of the FMRIB58_FA standard template could play a role due to the age discrepancy between the template subjects and the age of our subjects. Any resulting misalignment will consequently affect the skeletonisation and voxel-wise statistical analysis.

### Experiments

We demonstrated that it is possible to improve the alignment of DTI data by modifying the TBSS pipeline to use a group-wise atlas as the reference. We evaluated the performance and accuracy of different approaches in the registration step using a simulation study. The TBSS pipeline is intended to alleviate residual misalignment observed in methods like VBM, however this aim is not fully achieved using the ST and RS-TBSS approach (Figure 6). The three approaches studied all showed false-positive error in the specificity evaluation, defined as the finding of a significant difference between two groups when there was no true difference between them. The false positives were located in WM tracts that have been reported to be implicated in AD underlining the importance of controlling misalignment in the study of this disease. The spurious results in this specificity evaluation study may relate to misalignment, which occurred in the registration step and was not fully rectified during skeletonisation.

We showed that the group-wise method performed well at both detecting true positive results in the sensitivity evaluation study, and at not generating false positives in the specificity evaluation study.

The AD cohort in this study had a moderate degree of cognitive impairment at the time of scanning, with a mean (MMSE) score of 16/30 (standard deviation 5.5). Our results demonstrate that widespread changes in the microstructural integrity of white matter tracts are evident at this stage of the disease [32], [33], [34]. Assessing the relative contributions of grey matter atrophy and white matter tract degeneration to disease progression will be an important direction for future longitudinal work.

In this study there was no significant FA reduction found in the fornix and parahippocampal gyrus when the GW-TBSS method was used. These WM tracts showed significantly reduced FA in AD patients in this study when ST-TBSS and RS-TBSS were used, as well as in studies which employed these approaches [26], [30], [22], [23], [28], [27] and in our specificity evaluation study. The changes detected in these tracts in some studies but not others could be due to differences in the patients studied, however it is also possible that thin WM tracts such as the fornix are particularly vulnerable to misalignment errors.

Although the three conventional methods of registration used in TBSS produced similar mean FA maps and skeletons, there appeared to be greater variance in voxel-wise FA after registration when compared to the group-wise approach. In addition, the group-wise method resulted in significantly higher FA values from individual data sets projected onto the group skeleton after registration compared with the other approaches. This suggests that the group-wise method reduces the level of residual misalignment after the registration step. We believe that the present study is the first to demonstrate quantifiable improvements in DTI analysis through the use of a group-wise atlas in TBSS.

The group-wise registration has the advantage of avoiding the anatomical bias introduced by choosing a specific template in typical pairwise registration frameworks. Geng et al. compared group-wise registration with pair-wise group registration to reference, FMRIB58FA and most representative subject. They showed the group-wise registration reduces across-subject variation of FA images suggesting that the sensitivity in detecting white matter alterations between populations, as reflected by FA changes at a group level, should be improved by more accurate registration methods [35]. Further, Geng et al. applied the unbiased group-wise registration method on diffusion tensor images and the registered DTI images showed smaller shape differences in terms of reduced variance of the FA maps and more consistent tensor orientations [36]. However, incorporating a group-wise atlas into the TBSS pipeline has not been previously implemented and increasing number of studies on neurodegenerative disorders with cerebral atrophy necessitate the improvement of this whole-brain DTI analysis method. Future work might also extend the TBSS approach to use tensor information instead of FA in the registration step. Zhang et al. proposed an atlas construction method using the information encoded in tensors, especially the orientation information, which may enable more accurate alignment of fiber tracts [37]. This work has been presented as Tract-Specific Analysis (TSA) which blended the spatial skeleton and fiber approaches to perform group analysis [38].

Although the computational time required to generate a group-wise atlas is higher than ST-TBSS technique but lower than RS-TBSS, we believe that this is an important step to incorporate into studies where there are significant morphological differences between groups that could affect the registration process, such as in AD. In the case of an ongoing study, the process may be accelerated by registering new subjects to a previously created group-wise atlas [39] (given that there are enough patients and controls used to create the group-wise atlas to represent the variability in the population) and consequently to the standard space for further voxel-wise statistical analysis.

The group-wise atlas was created using FSL. However, this can be done using other registration methods such as NiftyReg (http://sourceforge.net/projects/niftyreg) [40], IRTK [41] or ANTS [42]. Further enhancement to the group-wise atlas can be made by employing tensor-based registration methods, such as DTI-TK [43], which can align white matter tracts better than FA-based approaches [44]. Other aspects such as interpolation and alternative cost functions remain important areas for further work. Interpolation has a smoothing effect on the resultant images and may introduce some error. In order to achieve better resampling accuracy, a high-order interpolation method could be used. Studying the precise effects of interpolation is an active research area but is beyond the scope of this paper. Therefore, in this paper trilinear interpolation was used in the TBSS pipeline. This choice requires no additional parameters to be set and was motivated largely by previous experience.

In conclusion, we have presented a further improvement to the TBSS pipeline by incorporating a group-wise atlas as the registration target. Our study suggests that the GW-TBSS is a promising method with improved reliability and reduced residual misalignment for examining the degeneration of white matter fiber tracts when the subjects in the study have cerebral atrophy and ventricular expansion as is commonly observed in aging and neurodegenerative disorders such as Alzheimer's disease.
